# Aging and separation from children: The health implications of adult migration for elderly parents in rural China

**DOI:** 10.4054/DemRes.2017.37.55

**Published:** 2017-12-07

**Authors:** Qian Song

**Affiliations:** 1 RAND Corporation, Santa Monica, USA. bravurasong@gmail.com

## Abstract

**BACKGROUND:**

Massive rural-to-urban migration in China has profoundly altered the family life of rural older adults, as adult children remain the primary caretakers of their elderly parents. And yet little is known about the health and well-being of the parents of adult migrants in rural China whose main source of support has been displaced.

**OBJECTIVE:**

This study takes a comprehensive view and compares the trajectories of self-rated health among the rural elderly and examines how these health trajectories are associated with adult children’s migration.

**METHODS:**

We analyze older adults aged 55 years and over in rural China, using four waves of data from the China Health and Nutrition Survey (1997, 2000, 2004, 2006) and multilevel growth curve models.

**RESULTS:**

The results show that parents of migrants persistently scored worse self-rated health across ages than their counterparts whose children had not migrated. Long-term migration of adults takes a heavier toll on the health of their elderly parents than short-term migration. However, these associations with children’s migration are driven by the migration of sons. The migration of daughters and of children of both genders may have disparate effects on the health trajectories of elderly men and women.

**CONCLUSIONS:**

The findings suggest that the interplay of gendered family dynamics and migration processes affects the health outcomes of older adults.

**CONTRIBUTION:**

The findings contribute to current debates on the health and well-being of family members left behind by migrants and call for further study of the relationship between migration and family processes in the well-being of migrant families.

## 1. Introduction

An increasing number of developing countries worldwide are witnessing intensified internal migration as a result of urbanization and industrialization. Among them, China is experiencing the largest migration in history. Since the 1980s, driven by market opportunities, a massive and increasing number of laborers from rural China have moved to urban areas as migrant workers. Between 2000 and 2010 the percentage of floating population (those residing outside of their county for six months or more) increased from 6% (78.8 million) to 17% (170.6 million) of the entire population (Liang and Ma 2004; Liang, Li, and Ma 2014). This massive migration has profoundly changed the lives and well-being of the families left behind in rural areas. Substantial efforts have been dedicated to understanding the health consequences of the left-behind children (Huang et al. 2016; Nguyen 2016; Ren and Treiman 2016; Tong, Luo, and Piotrowski 2015; Xu and Xie 2015) and spouses (Chen et al. 2015). However, in many of these societies, including rural China, health provision is largely lacking, and adult children remain the primary source of support for older adults. Little attention has been paid to the health and well-being of the parents of adult migrants, whose main source of support has been displaced.

With a growing older population impacted by their adult children’s migration, the well-being of the parents of migrants in rural areas has become a more pressing issue. In 2013 there were 202 million Chinese aged over 60, and it is projected that this number will reach 288 million by 2025 and 370 million by 2050 (National Statistics Bureau 2014; United Nations 2002). Today, approximately two-thirds of older adults reside in rural areas (China Statistics Press 2012). At least half of the rural older Chinese, an estimated 67 million people, are impacted by the migration of their adult children (China Statistics Press 2012; Peking University 2011). There is also a health gradient across rural and urban older adults: Rural older Chinese have much lower educational attainment and socioeconomic status (SES) levels than their urban counterparts. Rural communities also suffer from a lack of health services and profoundly unequal care provision (Shi 1993). As a result, rural mortality is nearly 30% higher than urban mortality, and the rural elderly are disadvantaged in various aspects of health, such as functionality and depression (Li et al. 2016; Stockman 2000; Wang et al. 2017; Zimmer, Kaneda, and Spess 2007; Zimmer, Wen, and Kaneda 2010).

The handful of literature examining the health of left-behind elderly in different social contexts has uncovered mixed results (Adhikari, Jampaklay, and Chamratrithirong 2011; Antman 2012; Levitt 1998). The current study draws on a national survey spanning nine years and presents a more comprehensive view of adult children’s migration and the health trajectories of left-behind parents. First, the analysis asks whether the self-rated physical health of elderly parents of adult migrants left behind in rural areas is worse than that of those with nonmigrant children. This analysis not only examines the association between children’s migration and parents’ health, but also how migration plays a role in the trajectories of health while aging. Considering that the parents of returned migrants and of nonmigrants experience different risk factors and that return migration may be driven by parents’ adverse physical health, resulting in reverse causality (Giles and Mu 2007), the analysis goes beyond the migrant–nonmigrant dichotomy to differentiate between parents with adult children currently engaged in migration and those with returned migrant children. This practice furthers previous research by specifying the role of adult children’s migration and disentangling reverse causality caused by return migration.

Second, the analysis addresses the role of the duration/intensity of migration among adult children from the parental household and their parents’ health trajectories. Most studies have differentiated migrants from nonmigrants without considering the heterogeneity in migration spells. Revealing such heterogeneity can further unpack the mechanisms of the migration process and familial outcomes. Finally, the analysis considers the relationship between children’s gender and parental health outcomes. In settings such as rural China, filial expectations are gendered (Lavely and Ren 1992). It is therefore likely that the association between children’s migration and elderly parents’ health differs according to the children’s gender.

It should be noted that due to data limitations, only migrant children living in the parental household are examined. However, of all adult children, the migration of adult children living in the household has the most profound impact on the well-being of the older adults because they are expected to provide primary instrumental, social, and emotional elderly care and have the most intensive interactions with the elderly parents. Also, the elderly are usually more financially dependent on adult children living in the household than on adult children who live elsewhere. Possible biases introduced by this practice are discussed in Section 7.

## 2. Background

**Figure 1:**
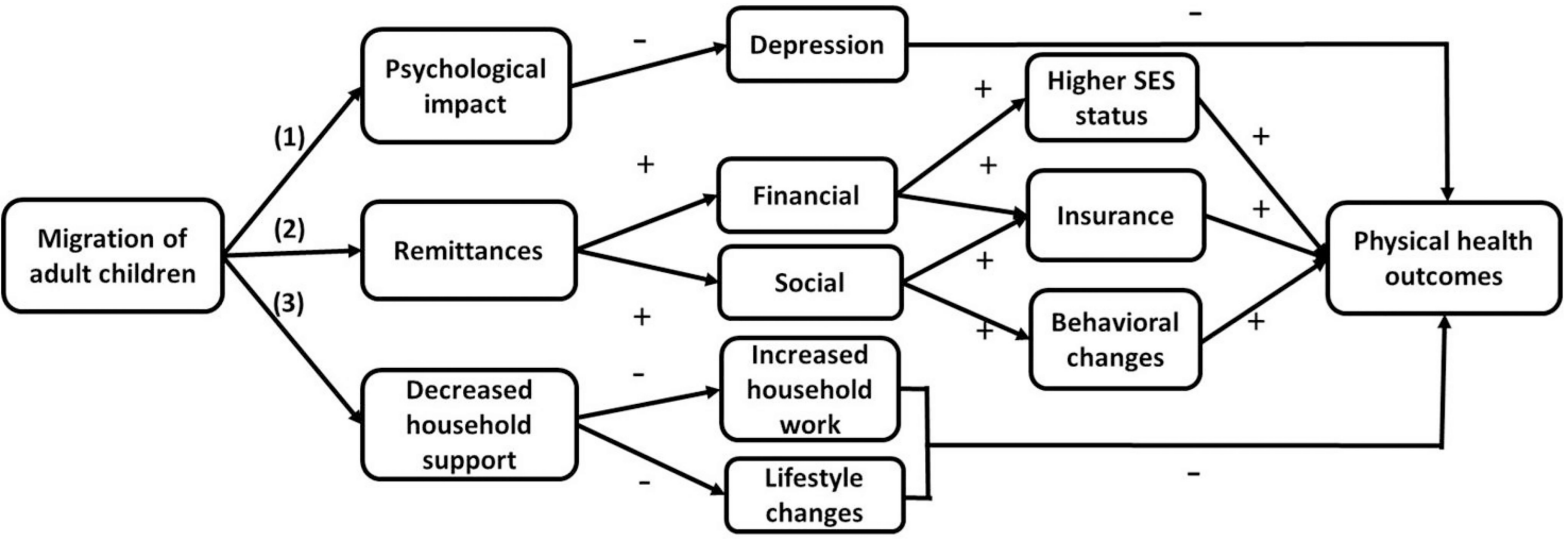
Conceptual pathways *Note:* A minus sign indicates a negative impact; a plus sign indicates a positive impact.

There are two theoretical frameworks that may help predict the relationship between adult children’s migration and the physical health of the elderly, the family disruption model and the new economics of labor migration (NELM) model. The principal pathways from migration of adult children to physical health outcomes of their elderly parents in rural China are summarized in Figure 1. The family disruption model, in particular the stress hypothesis, contends that family disruption produces disequilibrium and instability in family systems and creates stress in a variety of ways for both men and women: for example, economic stress or the stress associated with the premature assumption of social roles (pathways (1) and (3)) (Williams and Umberson 2004).

In rural China, where filial piety is particularly strong, adult children, especially adult sons, are expected to coreside or reside near to their elderly parents in order to provide assistance when needed (Giles and Mu 2007; Zhan and Montgomery 2003). The migration of adult children, especially those living with their parents, disrupts this family arrangement. As a result, parents of migrants are more depressed than other rural older adults (pathway (1)). This is probably due to the anxiety, stress, and/or loneliness associated with reduced local support from adult children (Song 2016). An increase in symptoms of depression is associated with older adults’ worse self-rated health (Mavaddat et al. 2011). Survey data also shows that children’s migration significantly reduces instrumental support received by elderly parents, and increases the time that the elderly spend on farm work and housework (Chang, Dong, and MacPhail 2011; Zuo and Li 2011). The resulting chronic stress and risk of incidents associated with working longer hours can be especially detrimental to the health of the elderly, who are at the greatest risk of disease and whose daily activity is limited (pathway (3)) (Cohen and Wills 1985). Golant (2015) defines ‘residential normalcy’ as the sense of comfort and mastery in the living environment of the elderly. When this sense of comfort is compromised through the migration of adult children the elderly may adopt riskier health behaviors such as alcohol use and smoking, or become more physically inactive for fear of being injured. Indeed, a detrimental effect of adult children’s migration on physical health has been found in several contexts, such as Mexico (Antman 2012). The family disruption model leads to the following expectation:

*Hypothesis 1a: The elderly with children who have migrated from the household have worse physical health than the elderly whose children have not migrated*.

However, the family disruption model does not consider the benefits that migration may bring to origin households. The new economics of labor migration (NELM) theory contends that the migration decision is often a mutually beneficial contractual arrangement between migrants and other household members (Stark and Bloom 1985). Several studies have found economic benefits of migration for rural households in China (see Rozelle, Taylor, and de Brauw 1999; Taylor, Rozelle, and de Brauw 2003). Remittances may reduce the financial stress on left-behind family members resulting from an overdependence on agricultural work. It has been found that left-behind elderly in rural China receive significantly more intergenerational financial transfers than the elderly who do not have migrant children (Song 2016; Zuo and Li 2011). This gives the parents of migrants better access to quality food, alleviates economic stress, and enables them to finance health insurance (pathway (2)). In addition, remittances received from migrant children allow a time-for-money exchange between the elderly and their nonmigrant children, in which the elderly transfer part of the remittance money to nonmigrant adult children living with them who provide eldercare (Frankenberg and Kuhn 2004).

Modern means of transportation and communication allow ongoing social and emotional support from migrant children (Knodel et al. 2010). Social remittances received from migrant children in the form of health-promoting knowledge and ideas may also change health behaviors among the elderly, improving their well-being through the increased probability of seeking medical care, using insurance, and visiting clinics (Adhikari et al. 2011; Levitt 1998). Consistent with this theory, several studies have found children’s migration to have positive effects in various settings, such as Bangladesh and Indonesia (Kuhn 2005; Kuhn, Everett, and Silvey 2011). Therefore, the prediction of the NELM model regarding the role of adult children’s migration differs from that of the family disruption model:

*Hypothesis 1b: The elderly who have children that have migrated from the household have better physical health than the elderly whose children have not migrated*.

### 2.1 Does duration of migration matter?

Both the family disruption model and the NELM model consider separation/migration duration to have an impact on left-behind parents’ health. The accumulative disadvantage theory argues that after a family disruption the stressors associated with the disruption pile up and lead to cumulative adversities that have long-term consequences. For example, studies have suggested that at older ages it takes a long time for the adverse effect of widowhood to manifest (Liu 2012). Similarly, the burdens of farm work and housework may accumulate over time, resulting in overload and harming physical health. A short-term separation from migrant children may not have such consequences. Therefore, we predict the following:

*Hypothesis 2a: Long-term migration of adult children from the parental household takes a heavier toll on, or benefits less, the physical health of elderly parents than short-term migration*.

By contrast, the NELM model suggests the opposite. While the ‘stress’ model posits that the disruption of families causes temporal decline in individual health because increased stress caused by transitory changes leads to adverse health outcomes (Liu 2012; Williams and Umberson 2004), the NELM model suggests that migration is part of a larger household strategy to better the well-being of individuals in the household. All adult children and children-in-law enter into a contractual eldercare arrangement, in which each of them decides on a shared duty of care, in terms of the kind and amount of support (Xu 2001). This change in support structure may initially create more stressful circumstances for the elderly. However, both theories predict that, as time goes by, the elderly will adjust to the new support arrangement. Therefore, the ‘stress’ model and the NELM model provide competing hypotheses regarding the duration of adult children’s migration:

*Hypothesis 2b: Long-term migration of adult children from the parental household takes a lesser toll on, or benefits more, the physical health of the elderly than does short-term migration*.

### 2.2 Does the gender of migrant children matter?

Finally, the disparity in gender roles assumed by sons and daughters in rural China suggests different adaptations – and predictions – of these theories. Rural China is patriarchal, patrilineal, and patrilocal, and the culturally prescribed ideal is that sons, rather than daughters, should provide care for the elderly (Xu 2001). Sons and their spouses, especially those living in the parental household, are the backbone of the elderlies’ safety net, and sons bear primary responsibility for financially supporting their parents in old age, even after marriage (Cong and Silverstein 2012). By contrast, daughters’ obligations are voluntary, usually performed out of love, and are more contextual and ambiguous than those of sons (Xu 2001; Zhan and Montgomery 2003). Gendered filial expectations remain strong in contemporary rural China (Cong and Silverstein 2012; Guo, Chi, and Silverstein 2009). Therefore, the migration of a son from the parental household is more likely to cause family disruption and to have a negative impact on the parents’ physical and mental health than the migration of a daughter. Although the support provided by a daughter-in-law can be beneficial, the absence of a son from the household defies the culturally ideal living arrangement and could take a toll on parental health (Silverstein, Cong, and Li 2006). Thus, when only sons migrate the outcome might be worse:

*Hypothesis 3: The elderly who only have sons that have migrated from their household will have worse physical health than the elderly whose children have not migrated*.

By contrast, the migration of daughters or couples from the household (usually a married son and his wife who migrate together) may be more consistent with the NELM model. Although coresident daughters may provide instrumental and social support to their elderly parents, Zuo and Li (2011) find that migrant daughters give approximately ten times more financial support than daughters who have not migrated, even though this is not expected of daughters. In addition, migrant daughters maintain greater contact with the origin household than migrant sons (Zuo and Li 2011). When couples migrate, they may send more remittances to compensate for their absence. This is especially true if their children remain behind in the grandparents’ household. In that case, resources are provided to compensate for the care and time provided by grandparents (Cong and Silverstein 2011). Therefore, we predict that:

*Hypothesis 4: The elderly whose single daughters or daughters accompanied by a son-in-law migrate have better physical health than those whose children have not migrated*.

It is also likely that the rewards and costs of having migrant children are different for the male and female parent. Elderly women in rural China are responsible for most of the housework and childcare, while men are expected to engage in more lucrative work (Chen 2004). When migrant children are absent, elderly women are likely burdened with increased household duties, whereas remittances sent home by migrant children could alleviate the stress on elderly men by reducing the necessity for them to work. Moreover, in a context in which the family is still women’s primary domain, family disruption may cause more stress to women than to men (Aneshensel and Pearlin 1987). However, elderly women are more economically dependent than elderly men (Zeng, Yuzhi, and George 2003), and the inflow of remittances from migrant children could reduce dependency on their spouses and help them obtain more economic freedom, thus contributing to their better health. The different exposure of elderly women and men to risk and protection warrants separate examination of these two groups.

## 3. Data

This study relies on the China Health and Nutrition Survey (CHNS). The CHNS is an ongoing open cohort longitudinal survey (1989–2011) with waves approximately every three years. The survey includes approximately 4,400 households with a total of 26,000 individuals, spanning all ages, in nine provinces (Liaoning, Heilongjiang, Jiangsu, Shandong, Henan, Hubei, Hunan, Guangxi, and Guizhou). These provinces span Eastern, Central, and Western China and vary in a number of characteristics, such as economic development and public resources. The sampling procedure uses a multistage random cluster process, and in each of the nine provinces a weighted sampling scheme based on income (low, middle, and high) was used to randomly select four counties. Whenever feasible, the provincial capital city and a lower income city were selected. Townships and villages within rural counties and urban/suburban neighborhoods within cities were selected randomly.

The CHNS is a household-based survey and follows the households belonging to the previous wave. All new households formed from the households sampled in the previous wave and residing in the sample areas were added to the subsequent wave. Attempts were made to conduct face-to-face interviews with each member of the chosen household. When a household member was not available, information was collected from other members knowledgeable about the indexed individual. Although the CHNS sample is not representative of the Chinese population, studies have shown that characteristics of the CHNS households and individuals are comparable to those shown in nationally representative samples (Chen et al. 2015; Short, Ma, and Yu 2000; Zimmer, Wen, and Kaneda 2010). The current study relies on the 1997, 2000, 2004, and 2006 waves because the question on the current migration status of each household member at the time of interview began in 1997, and self-rated health was collected up until 2006. We restricted our analysis to the elderly aged 55 and over who resided in rural communities as of the 1997 wave, and followed them to 2006. Loss to follow-up rate ranged from 1% to 9% across the waves, and 323 individuals had died by the end of 2006. In the panel an individual was observed more than three times, on average. In total, there are 3,983 person-year records included in the full analysis.

## 4. Measures

### 4.1 Self-rated health

Self-rated health is used as the dependent variable for its advantages in this context. Self-rated health has a “dual nature”: It is a “subjective and contextual self-assessment” and “an indicator of objective somatic and mental state, at one and the same time” (Jylhä 2009: 311). In many cultures, including China, self-rated health has proved to be a strong predictor of mortality (Idler and Benyamini 1997; Yu et al. 1998). Moreover, self-rated health can be a more inclusive measure than direct health indicators: It also captures direct bodily sensations, ailments, feelings, and other subtle bodily information that is not necessarily revealed in diagnosed diseases and functioning commonly used in medical research (Jylhä 2009). In the Chinese context it can be less biased than other measures, such as self-reported diagnosed diseases. In China there is a general underdiagnosis of chronic diseases due to underdeveloped public health provision and relatively low levels and poor quality of routine screenings and disease diagnosis^2^ (Qiao et al. 2008). This underdiagnosis is especially pronounced for rural older adults, who are generally much less educated, have lower SES, and are less likely to have medical insurance (Cheng et al. 2005; Yan et al. 2007). Therefore, self-rated health in this study is advantageous not only because it captures perceived health conditions but also because it is more accurate than self-reported diagnosed diseases.

In this analysis, self-rated physical health is based on the following survey question: “How would you describe your health compared to that of other people your age?” Responses ranged from 1 to 4, indicating excellent to poor health. The responses are reverse-coded so that higher scores indicate better physical health.

### 4.2 Parents of migrant children, returned migrant children, and nonmigrant children

The analyses focus on separation from adult children in rural areas through labor migration. The migration status of all household members is assessed at each time point in the survey via the question, “Is this person still in your household?” The individual is defined as a labor migrant if the option “No, sought employment elsewhere” is chosen. In this way, parents of current migrants are defined as those who have at least one child who has left the household to seek employment at the time of each interview. Parents of nonmigrant children are those who, from the first wave they were enrolled, had never reported a migrant child from their household at time of interview. In addition to these two groups, we further identify the parents of returned migrants. These are older adults who had migrant children in a previous wave but not in the current wave. If at least one of their children experienced another migration trip, they became parents of current migrants again. As an older adult can have multiple children with different migration profiles at each time of interview, the status of having current migrant children takes priority over having returned migrant children, which takes priority over having children who never migrated. To answer the first research question, the analysis compares parents of migrants, parents of returned migrants, and parents of nonmigrants, with parents of nonmigrant children as the reference group. All the migration-related variables are time-varying, and at each time point they are mutually exclusive.

To answer the second research question regarding the duration of children’s migration, the analysis divides parents of current migrants into two groups: children absent in one wave and children absent in two or more waves (see Chen et al. 2015 for an example of similar methods for categorizing migration duration using the CHNS). Therefore, in the second set of models there are four categories: parents of migrant children who have been absent in one wave, parents of children who have been absent in two or more waves, parents of returned migrant children, and parents whose children never migrated (i.e., parents of nonmigrants).

Similarly, to answer the third research question regarding the gender composition of migrant children and children-in-law, parents of migrants are divided into three categories: parents of male migrant children only, parents of female migrant children only, and parents who have migrant children of both genders. Ninety-nine percent of the parents of male migrant children only have migrant sons, with the remaining 1% having migrant sons-in-law in the household. Seventy-five percent of the parents of female children only have migrant daughters, with the remaining 25% having migrant daughters-in-law. Eighty-two percent of the parents who have migrant children of both genders have spousal migrants, and the remaining 18% have migrant daughters and sons. Thus, these categories can be roughly considered parents of migrant sons only, parents of migrant daughters only, and parents of spousal migrants. These are time-varying dummy variables, together with parents of returned migrants and parents of nonmigrants (reference).

### 4.3 Individual and household characteristics

The demographic characteristics of the older adults, their health behaviors, and resources available in the elderly household are controlled for. Demographic characteristics include gender, age (centered at the mean of 68 years old), and age-squared of the elderly. To measure older adults’ resources the analyses include a measure of their education (no formal education, primary school graduation, middle school graduation or higher), and a dummy variable to indicate whether they currently have medical insurance (yes/no). To adjust for health behaviors among the elderly the analyses also include time-varying dummy variables for whether they smoke or drink alcohol.

Other control variables reflect household characteristics. These include the presence in the household of the focal elderly parent’s spouse (yes/no), the presence of other adult children (yes/no), and the presence of preschool children (0–6 years old). These household contexts are important because they reflect the availability of familial social support, and potential care needs that the elderly might provide (Crimmins and Ingegneri 1990; Zimmer 2005). Region (northeast, coastal, inland) is another important control because it is associated with both the prevalence of labor migration and variation in self-rated reported health (Kondo et al. 2009). Finally, socioeconomic resources are measured through the household asset index and logged form of income per capita. The asset index comprises the ownership of a number of household consumer durables, with each item weighted according to number and approximate value (see Korinek et al. 2006 for a detailed description of this index using CHNS.) All demographic characteristics are time-invariant, and other covariates are time-varying.

It should be noted that the CHNS data does not include information on financial transfers from adult children, so it does not allow for the identification of the role of remittances. The analysis also compared results with and without household SES in the models. Household SES is not intended as a proxy for children’s transfers; however, it can be used to identify the role of the elderly households’ overall SES, partly via children’s financial transfers. Results are discussed in Section 7.

## 5. Methods

This analysis employs growth curve modeling techniques (Singer and Willett 2003) to investigate how the migration circumstances of adult children may shape typical self-rated physical health trajectories across age groups. Chen et al. (2015) provide a similar analytic approach using the CHNS. This analytic approach allows individuals to start with different levels of self-rated physical health. People may experience different rates of change in self-rated physical health as they advance in age. By allowing variation across individuals (i.e., random effects) the growth curve models take into account unknown heterogeneity across individuals, which is often the source of selection bias such as migration selection. Growth curve analysis can also handle unbalanced panel data. It is able to utilize all observations for the estimation of trajectories, regardless of the number of waves in which an individual is observed (Raudenbush and Bryk 2002). This has advantages over other regression models that disregard cases that later attrite. To further control for sample attrition bias and bias caused by the mortality of individuals, the analysis followed Chen et al. (2015) and entered two time-invariant dummy variables into each model to indicate both the deceased and attrition due to loss to follow-up in level 2 of the models. Since neither of the variables was statistically significant, they were dropped in the final models. All statistical analyses were performed with STATA 13 (StataCorp 2013).

The linear growth curve model can be specified as follows:

$${\text{SRH}}_{\text{ij}}=\pi_{0i}+\pi_{1i}({\text{Age}}_{\text{ij}}-68)+\pi_{2i}{\left({\text{Age}}_{\text{ij}}-68\right)}^{2}+{Z\prime }_{\text{ij}}A+\varepsilon_{\text{ij}}$$

$$\pi_{0i}=\alpha_{0}+{X\prime }_{0}B_{0}+\xi_{0i}$$

$$\pi_{1i}=\alpha_{1}+{X\prime }_{1}B_{1}+\xi_{1i}$$

$$\pi_{2i}=\alpha_{2,}$$

where SRH_ij_ is self-rated individual health i at wave j, and j ranges from 1 to 4 (1997, 2000, 2004, and 2006). Age_ij_ is the time metric variable and is the age of individual i at wave j. We centered age at 68 years old, the grand mean, to let the intercept reflect the level of self-rated health at the mean age of 68. π_0i_ and π_1i_ represent the i individual’s intercept and age slope - a random coefficient. We also included the quadratic age slope (7t2i), which was estimated as a fixed effect to take into account the nonlinear change of self-rated health with age. In predicting the intercept and age slope respectively at level 2, X’_0_ and X’_1_ indicate all time-invariant covariates, and B_0_ and B_1_ indicate the fixed effects. The vector of Z’_ij_ indicates all time-varying covariates, and A is its fixed effect. The term ε_ij_ is the level-1 residual, and the terms ξ_0i_ and ξ_1i_ are the individual-specific residuals (level-2 residual). We also incorporated the interaction of the time-varying migration variable with age and age squared in level 1 to examine how the role of children’s migration status varies by age. Because none of the interaction terms between migration variables and age squared are statistically significant, we excluded these terms in the final models.

It should be noted that migration selection and reverse causation could still play a role in the associations we observe. First, the adverse health condition of older parents could directly impinge on the migration decisions of their adult children, causing them to remain; or it could bring migrants back home, causing return migration (Giles and Mu 2007). Although this study identified return migrants and excluded them from the comparisons, it is still hard to determine whether or not potential migrants stayed because of a parent’s declining health. The ‘healthy migrant effect’ is another possible selection mechanism/process, where migrants are positively selected on their health status, so that their parents might share a latent genetic disposition for good health. These selections would lead to an underestimation of a negative effect, or an overestimation of a positive effect. However, compared to other methods, a pronounced advantage of growth curve modeling is that it is able to adopt a life-course perspective and capture a process – how the role of migration varies over time as individuals approach more advanced ages.

## 6. Results

Distribution of the sample by characteristics of rural older adults is shown in Appendix Table A-1. A comparison is made between key independent variables and self-rated health (SRH) as applied to the CHNS 2006 and to the China Health and Retirement Longitudinal Study (CHARLS) 2011, a nationally representative data set. We focus on rural older adults aged 55 and older. Appendix Table A-2 shows that these two samples are similar. Table 1 further displays characteristics of older adults by children’s migration status. Pairwise tests of difference were conducted for each pair of groups. Parents of migrants and returned migrants tend to have worse self-rated health than parents of nonmigrants. Compared to the parents of nonmigrants, parents of migrants and returned migrants tend to have worse self-rated health, higher education levels, a spouse present, to smoke and drink, to have fewer assets, and to reside inland, where the major migrant-sending communities are located. Parents of migrants are also less likely to live with other adult children and more likely to live with preschool children.

Results from growth curve models assessing the role of children’s migration status on parents’ self-reported health are shown in Table 2. The model for all older adults shows that when other factors are controlled for and parents of returned migrants are excluded, at age 68, older adults who have current migrant children report an average of 0.122 points worse health than those whose children have not migrated. When health behaviors are not controlled for, the disadvantage of the left-behind elderly declines by 6%, and when insurance status and household SES are not considered, such disadvantage further declines by 2% (table not shown). This suggests that both social and financial remittances may slightly help cushion the adverse effect of migration. The interactions between age and migration variables are not significant, suggesting that the migration of children is not associated with the rate of change in the health trajectories of older adults. The migration of children is similarly associated with the self-rated health of their older fathers and mothers.

**Figure 2:**
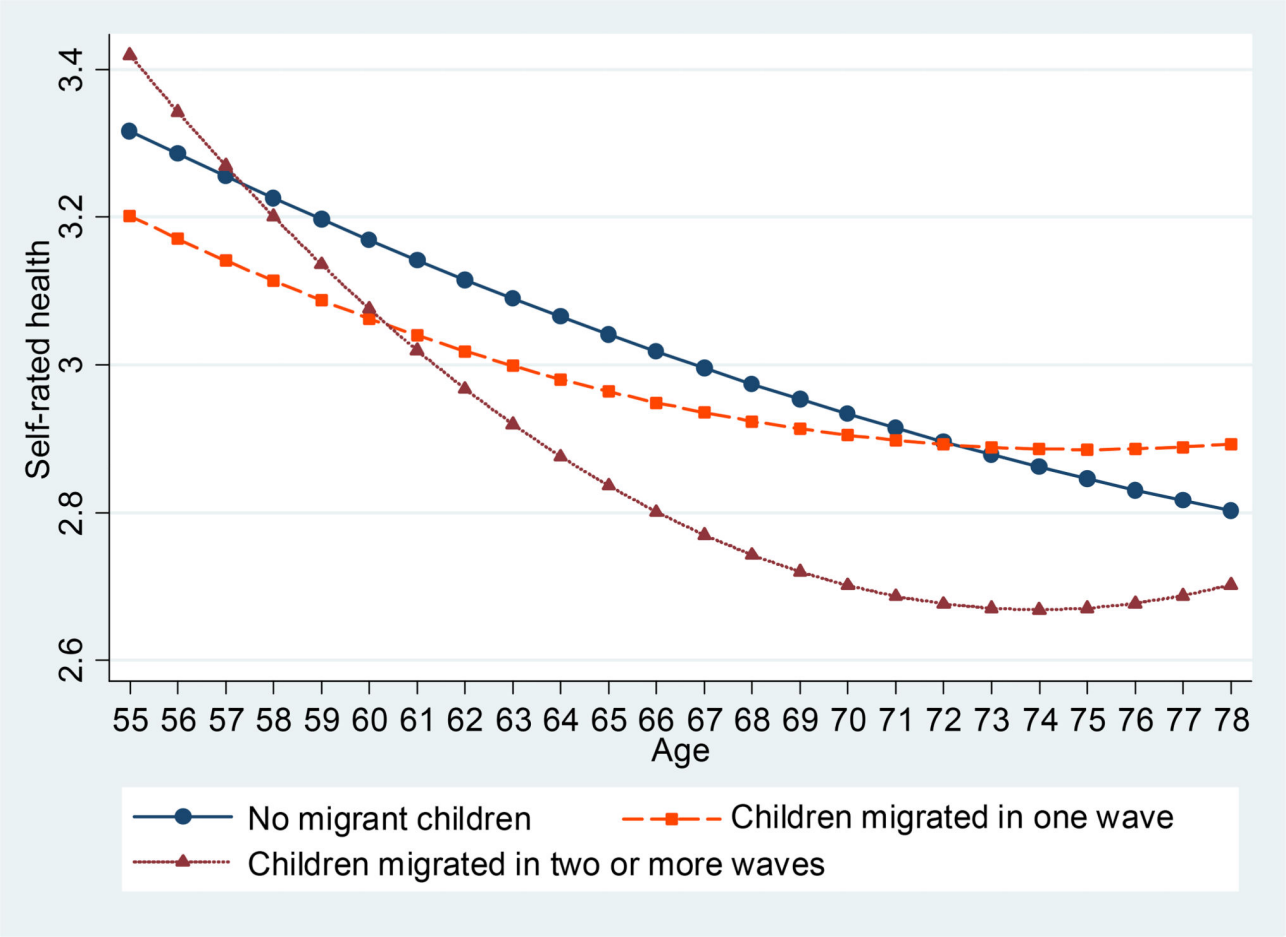
Predicted self-rated physical health trajectories, by children’s migration duration

Results in Table 3 further show that having children absent in two waves or more significantly reduces the self-rated physical health score by 0.183 points, whereas having children absent in one wave is not significantly associated with the health of their elderly parents. The predicted health trajectories of older adults by their children’s migration length are displayed in Figure 2. In a model that focuses on the parents of migrants only (not shown), having children absent in two waves or more significantly reduces their self-rated health by 0.14 points, compared with their counterparts whose children are absent in one wave.

**Figure 3:**
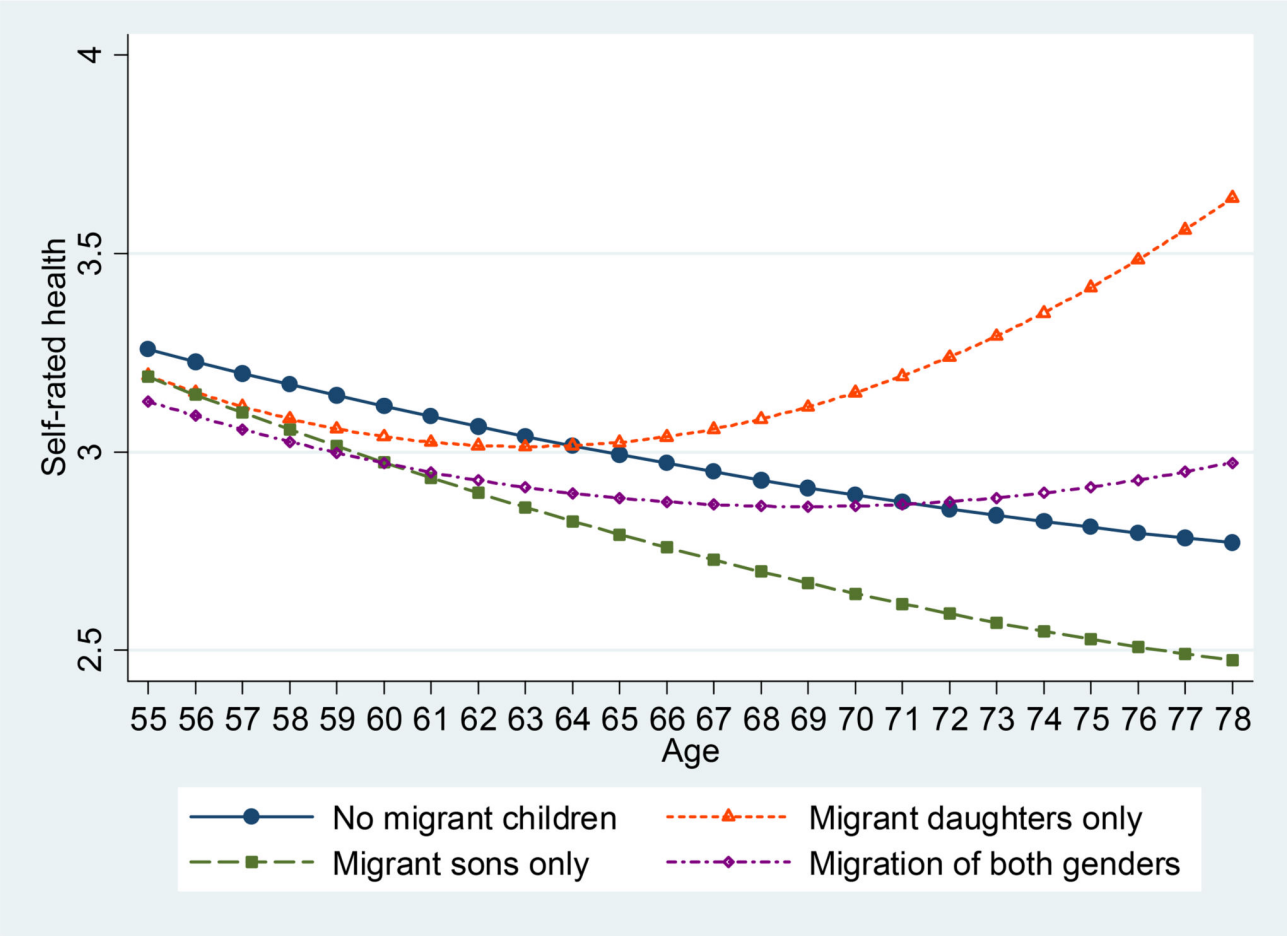
Predicted self-rated physical health trajectories, by gender composition of migrant children, elderly women

Finally, the analyses address the potential importance of migrant children’s gender for the health trajectories of parents left behind. Table 4 reveals that the ‘migration penalty’ observed previously is dominated by the migration of sons. The predicted health trajectory of older females according to the gender composition of their migrant children is displayed in Figure 3. In contrast to sons’ migration, as elderly women approach more advanced ages, having migrant daughters is increasingly associated with better self-rated health, though such a relationship does not exist for elderly men. It should be noted that in self-rated physical health a higher score does not necessarily indicate an improvement in health, but rather reflects a better evaluation of one’s own health compared to others around the same age. Parents of spousal migrants do not show a trajectory that is statistically significant compared to parents of nonmigrants. Elderly mothers of return migrants have declining health as they approach more advanced ages. This finding further suggests that not considering the timing of migration minimizes the role of migration in the health of the elderly parents.

## 7. Discussion

Migration of adult children is a distinct demographic process driven by globalization and urbanization, and it results in unique risks for the health of the elderly left behind as well as unique protections. It also provides an opportunity to investigate the interplay of the migration process and intergenerational exchange in the context of rural China.

The results suggest that, in general, the elderly parents of migrants have persistently worse self-reported health than parents whose adult children have not migrated (supporting Hypothesis 1a), and that long-term migration takes a heavier toll on the health of the elderly than short-term migration (supporting Hypothesis 2a). These findings are consistent with the family disruption model, which underscores the disruptive impact of the geographical separation of the elderly household from the adult children who are their normatively mandated caretakers, while the adverse associations of long-term separation support the cumulative disadvantage theory.

However, the disruption of the traditional intergenerational exchanges and the subsequent negative health consequences are dominated by the migration of sons only (supporting Hypothesis 3). The self-rated physical health trajectories of elderly women with migrant daughters are better than those of other older adults (providing some support for Hypothesis 4). While daughters are not normally expected to provide financial support to their older parents, migrant daughters actually send a similar amount of remittances as migrant sons. In addition, migrant daughters are able to maintain a high level of emotional support to their older parents by making frequent calls (Zuo and Li 2011). The support older parents receive from migrant daughters may exceed what is traditionally expected of a daughter. The older they get and the more fragile in health, the more important such support from migrant daughters is. A recent study in China (Zeng et al. 2016) found that daughters’ emotional support is more beneficial to older adults’ physical health than sons’, especially among the oldest-old. Moreover, daughters may maintain the close mother–daughter relationships expected by social norms (Cong and Silverstein 2012). As elderly women in rural China are frequently financially dependent on their spouses in a way that elder men are not (Zeng, Yuzhi, and George 2003; Zhan and Montgomery 2003), daughters’ financial support may benefit mothers by reducing such dependency on their spouse.

Further exploration of the analysis finds that this positive association is applicable to unmarried migrant daughters only. This suggests that as elderly women age, compared to married daughters and daughters-in-law that have migrated from the household, unmarried migrant daughters from the household have the advantage of more freedom and resources, which can benefit their mothers’ general physical health. However, it should be noted that this positive impact is only suggested and merits further investigation. For example, the positive association may have been overestimated due to the selection of unmarried daughters in the household into migration. Unmarried migrant daughters are more likely to be subject to a ‘healthy parent’ migration bias than sons. This is because unmarried daughters in the household are more likely than sons to stay and take care of their sick parents, and this selection renders the parents of migrant daughters from the household healthier.

Nevertheless, this research attempts to reduce migration selectivity. For example, our results show that the health of return migrants’ mothers declines with age, and this is not true for their fathers. Return migration selection may play a role in the case of father’s declining health, as his spouse usually assumes the role of caretaker. This minimizes the probability of migrants returning due to their father’s illness.

This study also sheds light on the pathways by which adult migration affects the health of elderly parents. In the Chinese context, these pathways appear more complicated than formerly thought. Most prominent is that although parents of migrants are more likely to smoke or drink, the associations between smoking and drinking behaviors and SRH are positive. These findings seem paradoxical, but they are consistent with findings from other studies in China, where drinking and smoking reflects better-off social status (Bonnefond and Clément 2014; Chen, Yang, and Liu 2010). Therefore, it is possible that the higher social status behind these unhealthy behaviors is positively associated with physical health. Further attempts to identify this path may benefit from information on the timing of behavior onset and quitting, and the quantity and frequency of these behaviors. Secondly, that household assets are positively associated with SRH suggests that the improved economic status of migrant parents helps cushion the adverse effects of migration. Because we do not have a direct measure of children’s transfer to parents, this finding is tentative.

The current study also has limitations. First and foremost, though there are advantages to growth curve modeling, it does not allow for a direct assessment of the causal relationship between migration and health. As discussed in detail earlier, this may underestimate a negative impact, or overestimate a positive effect. Secondly, the CHNS data does not include information on financial transfers from adult children, so it does not allow identifying the role of remittances. Thirdly, information on children not from the elderly household is not included. Although the migration of adult children from the household has the most profound impact on the well-being of the older adults, the associations derived from this study might have been less pronounced had we incorporated migrant children who were not from the elderly household. We also do not have data on adult children who live nearby and who can provide instrumental and other types of support. The availability of such support may reduce the probability of return migration caused by elderly parents’ serious health decline (Giles and Mu 2007). Fourthly, there are limited direct measures of emotional and social support provided across generations. Fifthly, because the migration status of adult children before Wave 1 was not observed, parents of return migrants may have been misidentified as nonmigrant parents in this wave, leading the results to underestimate the negative association of return migration and self-rated health. Finally, in the absence of a relevant variable, we are unable to reveal possible heterogeneities in elderly health resulting from children’s migration distance. Because periods of migration and migration distances are usually interconnected, we speculate that long- and short-distance migration may also be differentially associated with the health of elderly parents, though future research directly addressing the role of migration distance is needed.

In spite of these limitations, this study suggests that adult children’s migration, especially long-term migration, can have a detrimental effect on the health and well-being of their elderly parents in the place of origin. This is consistent with a previous finding that long-distance migration is associated with a higher probability of depression symptoms in elderly parents than short-distance migration (Song 2016). In light of these findings, stakeholders should develop policies that encourage industries to move job opportunities from the more developed coastal areas to the less-developed western and inland areas of China, where most migrant workers originate. As there is also a clear health gradient across regions, with the western and inland regions being more disadvantaged (Lu and Ran 2016), doing so would not only ease regional economic inequality but also contribute to reducing health inequalities across regions.

This study also highlights the fact that both migration and family processes should be considered when examining familial migration outcomes (Glick 2010). Solely focusing on family structure and living arrangements may be insufficient. The results have shown that even after controlling for family structure, various migration circumstances (e.g., migration spells) are differentially associated with the self-rated health of the elderly. On the other hand, the migration process is not a stand-alone factor that unidimensionally changes the well-being of the older adults. Rather, familial processes (e.g., gender roles in the family) are intertwined with migration processes and affect the health and well-being of elderly men and women in different ways. Looking ahead, as urbanization and globalization progress around the world, and especially in developing countries, migration processes may further engage with familial processes, and together change the well-being of the family members left behind. Future research integrating these processes is needed because these simultaneous processes could become an established way of family life in an increasing number of migrant-origin families.

## Figures and Tables

**Table 1:** Descriptive statistics, by children’s migration status

	Migrants	Returned	No migration	p
**Older-adult self-rated health**	2.25	2.23	2.33	b,c
**Older-adult demographics**				
Age	65.62	67.23	68.30	a,b,c
Female	0.49	0.45	0.53	c
No education	0.63	0.64	0.68	b
Primary school	0.23	0.18	0.19	b
Middle school or higher	0.14	0.18	0.13	c
**Older-adult living arrangement**				
Spouse present in household	0.80	0.82	0.76	b,c
Live with adult children	0.43	0.52	0.53	a, b
Live with preschool children	0.25	0.24	0.14	b, c
**Older-adult health behaviors**				
Smoke	0.32	0.32	0.27	b
Drink	0.34	0.30	0.27	b
**Older-adult resources**				
Have medical insurance	0.22	0.25	0.23	c
Household asset index	3.67	3.92	4.21	b
Logged household income in the	7.77	8.04	7.81	a,c
last year+1				
**Older-adult location**				
N ortheast region	0.01	0.01	0.07	b,c
East region	0.17	0.17	0.24	b,c
Inland	0.82	0.82	0.69	b,c
**N**	586	278	3,119	

*Note*:

a p<.05 comparisons made between those who had migrant children and returned migrant children,

b p<.05 comparisons made between those who had migrant children and who had no migrant children,

c p<.05 comparisons made between those who had returned migrant children and had no migrant children.

**Table 2:** Growth curve models of self-rated health of older adults, CHNS 1997–2006

	All		Females		Males	
	β	S.E.	β	S.E.	β	S.E.
**Children’s migration status**						
Migrated^a^	−0.122 **	0.041	−0.139 **	0.052	−0.107 *	0.051
Age, centered	−0.023 ***	0.003	−0.022 ***	0.004	−0.025 ***	0.004
Age, centered (squared)	0.000 *	0.000	0.001 *	0.000	0.000	0.000
Returned^a^	−0.091	0.056	−0.077	0.078	−0.095	0.068
Migrated × age	0.005	0.006	0.004	0.007	0.007	0.008
Returned × age	−0.007	0.009	−0.019	0.012	0.005	0.012
**Female**	−0.011	0.033				
**Primary school**^b^	0.015	0.034	0.006	0.059	0.037	0.044
**Middle school or higher**^b^	0.108 *	0.042	−0.045	0.083	0.170 **	0.050
**Older-adult living arrangement**						
Spouse present in household	−0.063 †	0.034	−0.063	0.043	−0.059	0.055
Live with adult children	0.014	0.033	0.004	0.042	0.026	0.041
Live with preschool children	−0.012	0.040	−0.014	0.049	−0.006	0.049
**Older-adult health behaviors**						
Smoke	0.089 **	0.033	0.016	0.073	0.107 **	0.036
Drink	0.154 ***	0.032	0.227 ***	0.063	0.130 ***	0.033
**Older-adult resources**						
Have insurance	−0.030	0.034	−0.008	0.046	−0.054	0.042
Household asset index	0.021 ***	0.005	0.018 **	0.007	0.024 ***	0.007
Logged household income in the last	−0.002	0.009	−0.006	0.012	0.004	0.013
year+1						
**Older-adult location**						
Northeast region^c^	0.133 *	0.066	0.075	0.090	0.205 **	0.078
East region^c^	0.130 **	0.040	0.109 *	0.049	0.160 **	0.049
**Constant**	2.221 ***	0.089	2.268 ***	0.104	2.136 ***	0.121
**Random effects – variance components**						
Level 1: Within-person	0.469 ***	0.144	0.491 ***	0.020	0.446 ***	0.018
Level 2: In linear growth rate	0.000 ***	0.000	0.000 ***	0.000	0.000 ***	0.000
Level 2: In intercept	0.080 ***	0.013	0.074 ***	0.017	0.079 ***	0.016
Log pseudolikelihood	−4439		−2335		−2093	
**N**	3,983		2,067		1,916	

Note:

† p<.1,

* p<.05,

** p<.01,

*** p<.001, ***p<.001,

a Reference is those who did not have migrant children,

b Reference is no schooling,

c Reference is inland region.

**Table 3:** Growth curve models of duration of children’s migration on self-rated health of older adults, CHNS 1997–2006

	All		Females		Males	
	β	S.E.	β	S.E.	β	S.E.
**Duration of children’s migration**						
Children absent in one wave^a^	−0.045	0.046	−0.057	0.058	−0.027	0.058
Children absent in two or more waves^a^	−0.183 **	0.059	−0.174 *	0.088	−0.203 *	0.082
Age, centered	−0.024 ***	0.003	−0.022 ***	0.004	−0.025 ***	0.004
Age, centered (squared)	0 **	0	0.001 *	0	0	0
Children absent in one wave × age	−0.082	0.055	−0.063	0.077	−0.092	0.068
Children absent in two or more waves × age	0.010	0.007	0.007	0.008	0.014	0.009
Returned^a^	0.002	0.011	0.005	0.015	−0.001	0.017
Returned × age	−0.007	0.009	−0.019	0.012	0.006	0.012
Constant	2.210 ***	0.089	2.249 ***	0.104	2.133 ***	0.121
**Random effects – variance components**						
Level 1: Within-person	0.469 ***	0.144	0.492 ***	0.020	0.443 ***	0.018
Level 2: In linear growth rate	0 ***	0	0 ***	0	0 ***	0
Level 2: In intercept	0.080 ***	0.013	0.075 ***	0.017	0.080 ***	0.016
Log pseudolikelihood	−4438		−2336		−2092	
**N**	3,983		2,067		1,916	

*Note:* This model also controls for older adults’ educational attainment, living arrangement, health behaviors, resources, and location.

* p<.05,

** p<.01,

*** p<.001, ***p<.001,

a Reference is those who did not have migrant children.

**Table 4:** Growth curve models of gender composition of migrant children on self-rated health of older adults, CHNS 1997–2006

	All		Females		Males	
	β	S.E.	β	S.E.	β	S.E.
**Gender composition of migrant children**						
Migrant sons only	−0.152 **	0.047	−0.201 **	0.062	−0.114 †	0.059
Migrant daughters only	0.138 †	0.083	0.211 †	0.121	0.071	0.102
Migration of both genders	−0.114	0.072	−0.087	0.092	−0.143	0.095
Age, centered	−0.023 ***	0.003	−0.022 ***	0.004	−0.025 ***	0.004
Age, centered (squared)	0 **	0	0.001 *	0	0	0
Migrant sons only × age	−0.005	0.006	−0.009	0.009	0.001	0.008
Migrant daughters only × age	0.034 *	0.015	0.04 *	0.017	0.022	0.02
Migration of both genders × age	0.022 †	0.012	0.019	0.018	0.024	0.016
Returned	−0.09	0.056	−0.084	0.077	−0.092	0.072
Returned × age	−0.009	0.009	−0.023 *	0.011	0.005	0.013
Constant	2.2 ***	0.054	2.206 ***	0.054	2.164 ***	0.069
**Random effects – variance components**						
Level 1: Within-person	0.467 ***	0.014	0.486 ***	0.02	0.443 ***	0.018
Level 2: In linear growth rate	0 ***	0	0 ***	0	0 ***	0
Level 2: In intercept	0.079 ***	0.013	0.075 ***	0.016	0.075 ***	0.017
Log pseudolikelihood	−4431		−2328		−2092	
**N**	3,983		2,067		1,916	

*Note*: This model also controls for older adults’ educational attainment, living arrangement, health behaviors, resources, and location

† p<.1,

* p<.05,

** p<.01,

*** p<.001, p<.001,

a Reference is those who did not have migrant children.

## Footnotes

2 For example, a study based on a nationally representative sample that collects biomarker information in China found that approximately 61.3% of men and 59.8% of women with diabetes had not previously been diagnosed (Yang et al. 2010).

## Appendix

**Table A-1:** Descriptive statistics on the characteristics of all older adults in the sample

	Means or proportions	Standard deviation	Min	Max
**Older-adult self-rated health**	2.31	0.77	1	4
**Children’s migration status**				
Did not migrate	0.86	0.35	0	1
Migrated	0.14	0.35	0	1
**By child’s duration**				
Children away in one wave	0.09	0.29	0	1
Children away in two or more waves	0.04	0.20	0	1
**By child’s gender**				
Son’s migration	0.09	0.32	0	1
Daughter’s migration	0.02	0.15	0	1
Migration of both genders	0.04	0.19	0	1
Returned from migration	0.07	0.25	0	1
**Older-adult demographics**				
Age	67.83	7.39	55	101
Female	0.52	0.50	0	1
No education	0.67	0.47	0	1
Primary school	0.19	0.39	0	1
Middle school or higher	0.13	0.34	0	1
**Older-adult living arrangement**				
Spouse present in household	0.77	0.42	0	1
Live with adult children	0.51	0.50	0	1
Live with preschool children	0.16	0.37	0	1
**Older-adult health behaviors**				
Smoke	0.28	0.45	0	1
Drink	0.28	0.45	0	1
**Older-adult resources**				
Have insurance	0.23	0.42	0	1
Household asset index	4.11	3.09	0	17
Logged household income in the last year+1	7.82	1.38	0	12
**Older-adult location**				
Northeast region	0.06	0.24	0	1
East region	0.23	0.42	0	1
Inland	0.71	0.45	0	1
**N**	3,983			

**Table A-2:** Comparisons of key characteristics of rural older adults aged 55+ in CHNS2006 and CHARLS2011

	CHNS2006	CHARLS2011
	Means or Proportions	Means or Proportions
**Older-adult self-rated health**^a^	2.17	2
**Children’s migration status**^b^		
Did not migrate	0.64	0.61
**Older-adult demographics**		
Age	71.55	71.53
Female	0.53	0.49
No education	0.72	0.67
Primary school	0.14	0.22
Middle school or higher	0.14	0.11
**Older-adult household structure**		
Spouse present in household	0.72	0.72
Live with an adult child^c^	0.43	0.33

Note:

a The scale of self-rated health used by CHNS2006 ranges from 1 to 4 (excellent, good, fair, poor). The scale of self-rated health closest to CHNS in CHARLS 2011 ranges from 1–5 (excellent, very good, good, fair, poor). Both scales are reverse coded so that higher scores indicate better health.

b Migrants in CHNS2006 are defined as individuals in the household who sought employment elsewhere at the time of survey. Migrants in CHARLS2011 are defined as individuals in the household who were away for over one month in the past year.

c A larger proportion of rural older adults lived with an adult child in CHNS2006 than that in CHARLS2011 (0.43 vs. 0.33). This difference can be partly attributed to the rapid increase in rural-urban migration between 2006 and 2011, which decreased the presence of adult children in rural elderly households.
